# Karyotype rearrangements and telomere analysis in *Myzus persicae* (Hemiptera, Aphididae) strains collected on *Lavandula* sp. plants

**DOI:** 10.3897/CompCytogen.v8i4.8568

**Published:** 2014-10-31

**Authors:** Mauro Mandrioli, Federica Zanasi, Gian Carlo Manicardi

**Affiliations:** 1 Dipartimento di Scienze della Vita, Università di Modena e Reggio Emilia, Via Campi 213/d, 41125 Modena, Italy

**Keywords:** Aphids, *Myzus persicae*, holocentric chromosomes, karyotype rearrangements, *de novo* telomeres, shelterin

## Abstract

Karyotype analysis of nine strains of the peach-potato aphid *Myzus persicae* (Sulzer, 1776), collected on *Lavandula* sp. plants, evidenced showed that five of them had a standard 2n = 12 karyotype, one possessed a fragmentation of the X chromosome occurring at the telomere opposite to the NOR-bearing one and three strains had a chromosome number 2n = 11 due to a non-reciprocal translocation of an autosome A3 onto an A1 chromosome. Interestingly, the terminal portion of the autosome A1 involved in the translocation was the same in all the three strains, as evidenced by FISH with the histone cluster as a probe. The study of telomeres in the *Myzus persicae* strain with the X fission evidenced that telomerase synthesised *de novo* telomeres at the breakpoints resulting in the stabilization of the chromosomal fragments. Lastly, despite the presence of a conserved telomerase, aphid genome is devoid of genes coding for shelterin, a complex of proteins involved in telomere functioning frequently reported as conserved in eukaryotes. The absence of this complex, also confirmed in the genome of other arthropods, suggests that the shift in the sequence of the telomeric repeats has been accompanied by other changes in the telomere components in arthropods in respect to other metazoans.

## Introduction

Karyotype features are usually stable within species, and chromosomal changes, if they occur, contribute to the formation of a post-zygotic barrier between biological populations causing the establishment of reproductive isolation and speciation as a possible consequence (Noor et al. 2001, Delneri et al. 2003, Kandul et al. 2007). Indeed, mating between individuals with different karyotypes frequently produces hybrids with a reduced fertility due to mis-segregation of homologous chromosomes during the first meiotic divisions (e.g. Kandul et al. 2007).

Despite these general rules, the speciation models were still problematic since numerous cases of intraspecific karyotype instability have been described in literature and at present the most extreme case was published by Lukhtanov et al. (2011) reporting in the butterfly *Leptidea sinapis* (Linnaeus, 1758) the first clearly documented example of explosive chromosome number evolution through intraspecific and intrapopulation accumulation of multiple chromosomal changes. At the same time, the hybrid-sterility model is controversial in some taxa (as revised by Faria and Navarro 2010) so that its true plausibility is difficult to evaluate.

Aside from special cases, such as polyploidy, chromosomal speciation remained a controversial mechanism, especially in animals other than mammals (e.g., Coyne and Orr 2004), since up till now few studies have systematically analyzed the number of chromosomal rearrangements between taxa as a function of the divergence time measured molecularly (Coyne and Orr 2004). An intriguing exception is represented by the large genus *Agrodiaetus* (Hübner, 1822) (Lepidoptera: Lycaenidae), which exhibits an unusual interspecific diversity in chromosome number, from n = 10 to 134, allowing to assess that a rapid karyotypic diversification is likely to have contributed to this explosive speciation rate (Kandul et al. 2007).

The peach potato aphid *Myzus persicae* (Sulzer, 1776) is a good experimental model for the study of chromosome rearrangements since numerous variations regarding both chromosome number and structure have been reported (Blackman 1980, Lauritzen 1982, Rivi et al. 2012; Monti et al. 2011, Monti et al. 2012a, Monti et al. 2012b, Kati et al. 2014). Several populations of *Myzus persicae* were, for example, heterozygous for a translocation between autosomes 1 and 3 and this rearrangement is involved in the resistance to organophosphate and carbamate insecticides (Spence and Blackman 1998). *Myzus persicae* populations with 13 chromosomes have also been identified in various countries as the result of independent and diverse fragmentations of the autosome (A) 3 suggesting that different naturally occurring rearrangements of the same chromosomes may be observed in the aphid karyotype (Blackman 1980, Lauritzen 1982; Monti et al. 2012a, Monti et al. 2012b, Rivi et al. 2012). Lastly, some *Myzus persicae* clones possessed an intra-individual mosaicism, mainly involving fissions of chromosomes A1, A3 and X (Monti et al. 2012a, Kati et al. 2014).

The evolutionary history of the *Myzus persicae* group is marked with speciation events (for a review see Blackman and Eastop 2007) and the tobacco specialist subspecies *Myzus persicae nicotianae*, known as the tobacco aphid, is a notable example since it preserved its genomic integrity through time and across a wide geographical scale by investing in asexual life cycle in most parts of the world (Blackman 1987, Margaritopoulos et al. 2007a, 2007b, 2009).

The frequent occurrence of different chromosome numbers and the inheritance of chromosomal fragments have been related to the holocentric structure of aphid chromosomes (Mandrioli and Manicardi 2012, Manicardi et al. 2014), since chromosomal fragments can contact the microtubules and move properly in the daughter cells during cell division so that they are mitotically stable (Blackman 1980). However, the molecular machinery involved in such rearrangements is still not clarified and the holocentric nature of chromosomes may explain the inheritance of rearranged chromosomes, but not their origin.

The spread of chromosomal rearrangements has also been favoured in *Myzus persicae* by the continuous expression of the telomerase gene, which allows a *de novo* synthesis of new telomeres at the chromosomal breakage sites (Monti et al. 2011) and by the fast aphid reproduction based on apomictic parthenogenesis (Manicardi et al. 2014). This aspect is particularly intriguing considering that parthenogenesis has been described in bdelloid rotifers as a mechanism favouring speciation since it forces the reproductive isolation (D. Fontaneto, personal communication).

As Loxdale et al. (2011) mentioned in their review about specialization in animals, *Myzus persicae* could be an ideal experimental model to analyze rapid evolution, i.e. measured in perceptible time scale, since the agricultural practices could act as a strong selection pressure favouring evolutionary changes over short periods.

In the present paper we analysed the presence of karyotype variants in nine *Myzus persicae* strains collected on *Lavandula* sp. plants. Moreover we verified if the synthesis of *de novo* telomeres is common in *Myzus persicae* populations with fragmented chromosomes and analysed the evolutionary conservation of the shelterin complex, a group of proteins generally associated with telomere functioning.

## Material and methods

Specimens of *Myzus persicae* were obtained from 9 different aphid populations collected on *Lavandula* sp. plants. In particular, the strains labelled as Mo1, Mo2, Mo3 and Mo4 have been collected in Modena (Italy), whereas the strains Re1, Re2a, Re2b, Re3 and Re4 have been collected in Correggio (Reggio Emilia, Italy). Each population was established as a clone from a single female aphid originally collected from the field and thereafter maintained as a colony of parthenogenetic females on pea (*Pisum sativum*, Linnaeus, 1758) plants at 19 °C with a light-dark regime of 16 hours light and 8 hours darkness.

Chromosome preparations were obtained from parthenogenetic females by spreading embryo cells, as reported by Mandrioli et al. (1999) In order to analyse chromosome number, slides were stained with a 100 ng/ml propidium iodide solution in phosphate buffer for 15 minutes at room temperature. For each different karyotype, measurements of chromosome length were performed on 50 metaphases using the software MicroMeasure, available at the Biology Department at Colorado State University website (http://rydberg.biology.colostate.edu/MicroMeasure).

DNA extraction, following a standard phenol-chloroform protocol, and fluorescent *in situ* hybridization (FISH) have been described in Mandrioli et al. (1999).

The 28S rDNA genes have been amplified using the primers F (5’-AACAAACAACCGATACGTTCCG) and R (5’-CTCTGTCCGTT TACAACCGAGC), designed according to the insect 28S rDNA sequences available in GenBank. Amplification was performed using a Hybaid thermal-cycler at an annealing temperature of 60 °C for 1 minute (min) with an extension time of 1 min at 72 °C.

In order to amplify a DNA sequence containing the complete aphid histone gene cluster, the primers HIS-CLUST-F (5’-cgaaaccgtaaagggtacga) and HIS-CLUST-R (5’-ggcggctttgactttattga) have been designed on the basis of the *Acyrthosiphon pisum* genomic scaffold 368 (NW_003383857.1, from base 259987 to 272662). The amplification of a 7379 bp fragment was carried out by an Hybaid thermal-cycler using the Fermentas Long PCR Enzyme Mix making annealing and extension at 68 °C for 8 min for 25 cycles, according to the manufacturer’s instructions.

PCR digoxigenin labelling of the subtelomeric repeat was performed with a PCR DIG labelling kit according to the Roche protocol using the specific oligonucleotide primers MpR-F (5’–TCAAAGTTCTCGTTCTCC–3’) and MpR-R (5’–GTTTTAACAGAGTGCTGG–3’), designed according to the subtelomeric repeat sequence available in the literature (Spence et al. 1998). The reaction conditions were 94 °C for 90 sec (denaturation), a total of 25 cycles of 94 °C for 30 s, 51 °C for 30 sec (annealing) and 72 °C for 30 sec (extension), and with a final extension step at 72 °C for 7 min.

In order to localize the telomeric (TTAGG)*_n_* repeats, a probe was obtained by PCR amplification using the two primers F (TTAGG)_5_ and R (CCTAA)_5_ in the absence of temfig, as described by Ijdo et al. (1991).

Random priming probe biotin-labelling was performed with the Biotin High Prime (Roche), whereas the PCR digoxigenin labelling were performed using the Dig High Prime (Roche). Both labelling were done according to the Roche protocols.

Propidium-stained and FISH slides were observed using a Zeiss Axioplan epifluorescence microscope. Photographs of the fluorescent images were taken using a CCD camera (Spot, Digital Instrument, Madison, USA) and the Spot software supplied with the camera and processed using Adobe Photoshop (Adobe Systems, Mountain View, CA).

Bioinformatic analyses for homologous genes coding for the proteins POT1, TRF1, TRF2, RAP1, TPP1 and TIN2 in aphids and other arthropods have been performed by BLAST alignments in GenBank (http://blast.ncbi.nlm.nih.gov/Blast.cgi) both at DNA and protein levels using different homologous genes as reference sequences (Table 1). A further search has been performed by BLAST alignments in aphids at AphidBase (http://www.aphidbase.com).

**Table 1.** GenBank sequences used for bioinformatic comparative analyses.

Telomere-associated proteins	Orthologous proteins in GenBank
POT1	*Homo sapiens* (AAH02923), *Schizosaccharomyces pombe* (CAB16192)
TRF1	*Homo sapiens* (NP_059523), *Schizosaccharomyces pombe* (NP_595979)
TRF2	*Homo sapiens* (NP_005643)
RAP1	*Homo sapiens* (ABA64473), *Schizosaccharomyces pombe* (BAB70735)
TPP1/TEBPα	*Danio rerio* (NP_001124265), *Stylonychia lemnae* (AAU95535)
TIN2	*Homo sapiens* (AF195512)

## Results

**Figure 1.**
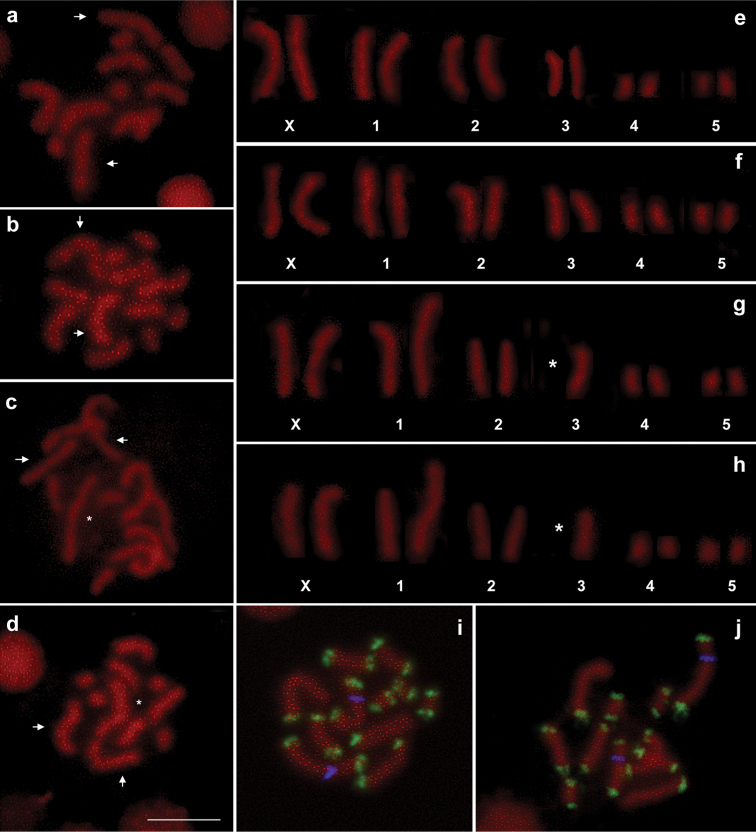
Chromosomal figs (**a–d**) and karyotypes (**e–h**) obtained from specimens belonging to clones Mo1 (**a**, **e**) e Mo2 (**b**, **f**), Mo3 (**c**, **g**) and Mo4 (**d**, **h**). Simultaneous *in situ* hybridization with the histone (in blue) and subtelomeric DNA probes (in green) (**i**–**j**) revealed in both clones Mo3 (i) and Mo4 (**j**) that the A1–A3 translocation involved the autosome 1 telomere close to the histone probe. Arrows indicate X chromosomes; asterisks indicate rearranged autosomes. Bar = 10 mm.

**Figure 2.**
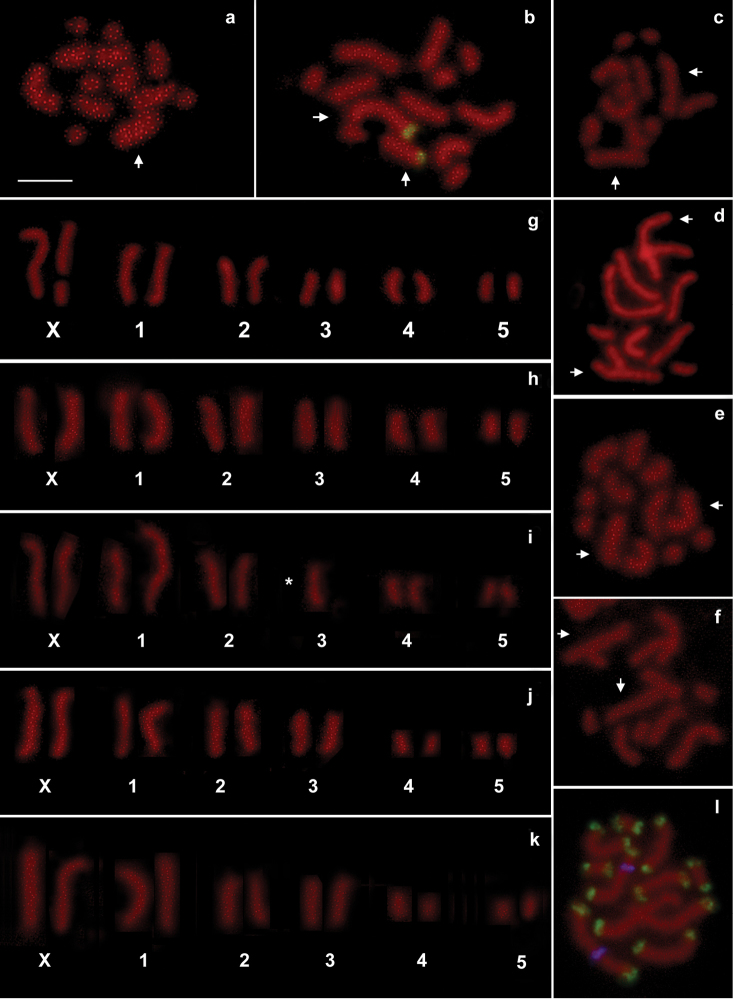
Chromosomal figs (**a**, **c–f**) and karyotypes (**g–k**) obtained from specimens belonging to clones Re1 (**a**, **g, e**) Re2a (**c**, **h**), Re2b (**d**, **i**), Re3 (**e**, **j**) and Re4 (**f**, **k**). Fluorescent *in situ* hybridization with the 28S probe (in green) (**b**) revealed that the fragmentation at the X chromosome in clone Re1 occurred at the telomere opposite to the NOR (**b**). Simultaneous *in situ* hybridization with the histone (in blue) and subtelomeric DNA probes (in green) in clone Re2b (**i**) revealed that the A1–A3 translocation involved the autosome 1 telomere close to the histone cluster. Arrows indicate X chromosomes. Asterisks indicate rearranged autosomes. Bar = 10 mm.

The standard karyotype of *Myzus persicae* parthenogenetic females consists of 12 chromosomes (five pairs of autosomes and two X chromosomes) (Mandrioli et al. 1999). The analysis of the strains collected on *Lavandula* plants showed chromosome numbers ranging from 2n = 11 to 2n = 13. In particular, Mo1 (Fig. 1a, e), Mo2 (Fig. 1b, f), Re2a (Fig. 2c, h), Re3 (Fig. 2e, j) and Re4 (Fig. 2f, k) showed a standard 2n = 12 karyotype, whereas Mo3 (Fig. 1c, g), Mo4 (Fig. 1d, h) and Re2b (Fig. 2d, i) have a chromosome number 2n = 11 due to the non-reciprocal translocation of an autosome A3 onto an A1 chromosome.

Previous study showed that the histone cluster map eccentrically on the autosome 1 (Mandrioli and Manicardi 2013), so that double *in situ* hybridization with the subtelomeric DNA repeat and the histone cluster as probes indicated that the non-reciprocal translocation observed in Mo3 (Fig. 1i), Mo4 (Fig. 1j) and Re2b (Fig. 2l) strains occurred at the same telomere of the autosome 1. Furthermore, propidium iodide staining revealed that the strain Re1 (Fig. 2a, g) has 2n = 13, as a consequence of a fragmentation of a single X chromosome involving the telomere opposite to the NOR-bearing one, as evident after FISH with the 28S probe (Fig. 2b).

**Figure 3.**
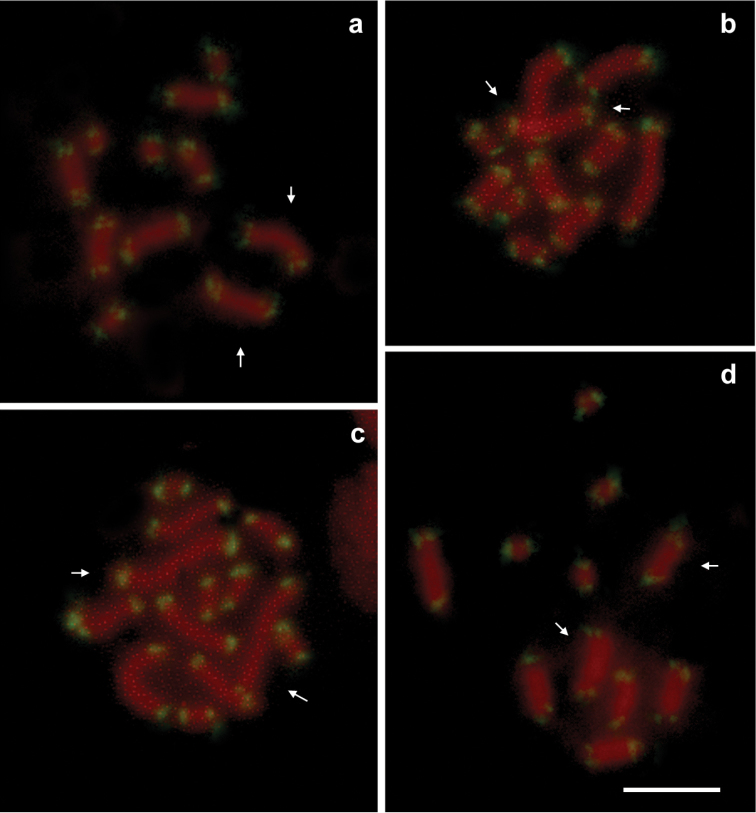
FISH with the telomeric (TTAGG)*_n_* probe showed evident telomeric repeats at each chromosomes in clones Mo3 (**a**), Mo4 (**b**), Re1 (**c**) and Re2b (**d**). No interstitial telomeric signals were present in clones Mo3 (**a**), Mo4 (b) and Re2b (**d**) possessing a chromosomes derived from an autosome A1–A3 fusion. All telomeres resulted labelled by the (TTAGG)*_n_* telomeric probe in clone Re1 including the X chromosome involved in the fission suggesting that a *de novo* telomere synthesis occurred in this clone (**c**). Arrows indicate X chromosomes. Bar = 10 mm.

**Figure 4.**
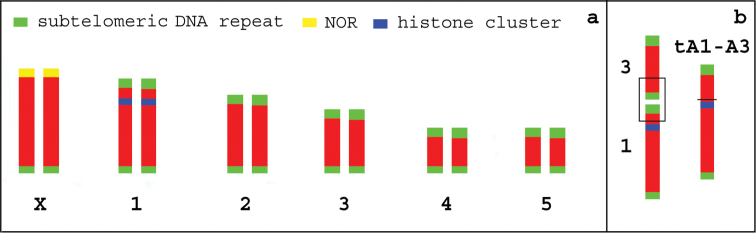
According to previous studies (Spence et al. 1998, Mandrioli and Manicardi 2012), the standard karyotype of *Myzus persicae* females consists of five couples of autosomes and two X chromosomes, whose rearrangements can be studied using different chromosomal markers (the subtelomeric DNA repeat, the NOR regions, and the histone cluster) (**a**). The analysis of karyotypes of clone Mo3, Mo4 and Re1 indicated that the observed A1-A3 translocation involved the A1 telomere near to the histone cluster an resulted in the loss of both the subtelomeric and the telomeric sequences (**b**).

Interestingly, in the clone Re1 all telomeres resulted labelled by the (TTAGG)*_n_* telomeric probe including the X chromosome (and its fragment) involved in the fission suggesting that a *de novo* synthesis of telomeres occurred in this clone (Fig. 3c). No interstitial telomeric signals have been observed in clones Mo3, Mo4 and Re2b possessing a fusion between a copy of autosomes A1-A3 (Fig. 3a, b, d). This result indicated that the A1-3 translocation also involved the loss of both the telomeric and subtelomeric sequences originally present at the chromosomal termini involved in the translocation site, as highlighted in the karyogram drawn in Fig. 4.

Taking into account that the unique aphid protein studied regarding the telomere functioning has been the telomerase (Monti et al. 2011), a survey for orthologues of the proteins consituting the shelterin complex has been performed in the genomes of the aphids *Acyrthosiphon pisum* (Harris, 1776) and *Myzus persicae* by BLAST alignments both at DNA and protein levels (Table 1), but no orthologues have been found for genes/proteins POT1, TRF1, TRF2, RAP1, TPP1 and TIN2. Similar results have also obtained in the insects *Tribolium castaneum* (Herbst, 1797) (order Coleoptera), *Apis mellifera* (Linnaeus, 1758) (order Hymenoptera), *Anopheles gambiae* (Giles, 1902) (order Diptera) and *Bombyx mori* (Linnaeus, 1758) (order Lepidoptera) and the mites *Tetranychus urticae* (Koch, 1836) and *Varroa destructor* (Anderson & Truman, 2000) assessing that genes coding for the shelterin proteins are not present in all the currently available arthropod genomes.

## Discussion

Holocentric chromosomes have been frequently described as a powerful tool to stabilize and inherit chromosomal mutations resulting in karyotype changes (Monti et al. 2012a, 2012b). Even if it is not clear if the observed karyotype variants have phenotypic effects over short temporal and spatial scales on aphid evolution and adaptation, the presence of chromosomal fissions and fusions (together with holocentrism and a constitutive expression of telomerase) could allow a rapid karyotype evolution at ﬁne geographic scales so that aphid species could be the sum of populations that can have different karyotypes that in turn can give diverse genetic/ecological/evolutionary responses in relation to imposed selective environmental forces (Monti et al. 2012a, 2012b). Indeed the ﬁne-scale patchwork of chromosome rearrangements observed in aphids could increase their potential for local adaptation and differentiation for instance on different host plants that could also explain the success of *Myzus persicae* as a polyphagous pest crop species.

The identification of several *Myzus persicae* populations bearing rearranged karyotypes made this species a complex, but intriguing, model for the study of aphid cytogenetics (Lauritzen 1982, Blackman 1987, Fenton et al. 1998, Spence and Blackman 1998, Loxdale 2007, Monti et al. 2012a, 2012b, Manicardi et al. 2014).

In this paper we report the presence of rearranged karyotypes, including fissions and translocations, in *Myzus persicae* strains collected on *Lavandula* plants. The analysis of their karyotypes confirmed that autosomes 3 and 1 are the chromosomes mostly involved in changes in the *Myzus persicae* complement (Rivi et al. 2012, Monti et al. 2012a, Kati et al. 2014) and supported previous results suggesting that also the X chromosome can be fragmented (Monti et al. 2012a, 2012b).

Previous literature data (Monti et al. 2012a, 2012b, Kati et al. 2014) highlighted that most of the rearranged karyotypes has been observed in aphid clones collected on tobacco plants, where the stability of the karyotype can be influenced by the clastogenic effects of nicotine (Trivedi et al. 1990, 1993, Sen et al. 1991, Arabi 2004, Sassen et al. 2005). Similarity to nicotine, also the linalyl acetate (one of the major components of the lavender oil) has a genotoxic effect in mammalian cells, where it induced the formation of micronuclei (Di Sotto et al. 2011) so that we could hypothesize that this compound could be at the basis of the chromosomal fragmentations described in this paper. Interestingly, not all the aphid strains collected on *Lavandula* plants showed rearranged karyotypes suggesting that *Myzus persicae* populations on *Lavandula* plants could consist of strains with a different capacity to metabolize the linalyl acetate in other compounds (such as the linalool) without any genotoxic activity (Di Sotto et al. 2011).

The fission of chromosomes by tobacco and lavender oil mutagens may be lethal in organisms with monocentric chromosomes (possessing a localized centromeres), since chromosomal fragments tend to be lost during mitosis and meiosis. By contrast, aphids can cope with this due to the holocentric nature of their chromosomes. As a consequence, chromosome fragments can move to the daughter cells at successive cell divisions.

Our results confirmed that some portions of the aphid chromosomes seem to be more prone to fragmentation than others in presence of potential genotoxic compounds. Indeed, a fragmentation of the X chromosome similar to that reported in the present paper has been described in other *Myzus persicae* strains and it was localized near (or within) the heterochromatic band enriched in satellite DNAs (Monti et al. 2012a, 2012b). The presence of chromosome breakpoints occurring within constitutive heterochromatin is well established in the scientific literature and, for instance, much of the evolution of mammals and some insects (such as grasshoppers) involved pericentromeric heterochromatin that is known to be particularly variable (John 1983, Blackman et al. 2000). *Myzus persicae* autosome A3 is involved in a heterozygous translocation on an autosome A1 in three *Lavandula* strains further supporting the suggestion that translocations between these autosomes are frequent. Indeed, the same translocation has been previously found in two Greek clones collected on tobacco plants (Kati et al. 2014) and a variant consisting in a partial reciprocal translocation between the A1 and A3 has been reported to have a worldwide distribution (Blackman 1980, Blackman et al. 2007).

Our data showed that the A1-A3 fusion seems to involve always the same terminal end of the autosome 1. Previous experiments (Mandrioli et al. 2014) reported that the terminal portions of autosomes 1 and 3 are in tight proximity in *Myzus persicae* interphase nuclei suggesting that their proximity could favour their fusion resulting in reciprocal and/or non-reciprocal translocations. The presence of recurrent chromosomal rearrangements in *Myzus persicae* could therefore be related to the specific architecture of the aphid interphase nucleus.

From a chromosomal point of view, the species *Myzus persicae* is the sum of populations that have different karyotypes. Interestingly, similar karyotypic variants have been identified on different host plants (Monti et al. 2012a, 2012b, Rivi et al. 2012, 2013, Kati et al. 2014) suggesting that no host-specific karyotype are present in this species with the exception of *Myzus persicae nicotianae* on tobacco (Blackman 1987).

A further element of interest in the *Lavandula* clones is related to their ability to synthesize new telomeres after chromosomal breakages. In aphids, telomeres consist of stretches of the (TTAGG)*_n_* repeat. This simple sequence has been reported also in the majority of insects (Sahara et al. 1999, Mandrioli 2002, Frydrychová et al. 2004, Vitkova et al. 2005, Monti et al. 2011, Kuznetsova et al. 2012) and in other arthropod groups (sea spiders, chelicerates, myriapods, and crustaceans) (Traut et al. 2007), and seems to be ancestral for the phylum Arthropoda (Lukhtanov and Kuznetsova 2010). However, the ancestral (TTAGG)*_n_* telomeric motif has been repeatedly lost or replaced with other sequences during insect evolution (Vitkova et al. 2005, Mravinac et al. 2011, Mandrioli et al. 2012, Gokhman et al. 2014). For example, in the clade Antliophora (Diptera, Siphonaptera and Mecoptera) the canonical telomeres have been replaced by long repeated sequences, as reported in the non-biting midge *Chironomus pallidivittatus* (Malloch, 1915) (Zhang et al. 1994), or by the HetA and TART retrotransposons, as occurred in the fruit fly *Drosophila melanogaster* (Meigen, 1830) (Pardue and DeBaryshe 2003).

Differently from the extensive study of the telomere composition, few papers have been focussed on the proteins associated to the telomere functioning in insects, with the exception of *Drosophila melanogaster*, where telomeres are capped by a complex of fast-evolving proteins, called terminin (Raffa et al. 2011). However, none of the terminin proteins is evolutionarily conserved outside the *Drosophila* genus suggesting that flies rapidly evolved terminin to bind chromosome ends in a sequence-independent fashion probably slightly before the loss of the canonical insect telomeres (Raffa et al. 2011).

In mammals, telomeres are capped by different proteins that play vital roles in telomere length regulation and chromosomal end protection (Giannone et al. 2010). In particular, a relevant role in the mammalian telomeres is played by shelterin, a six subunit complex composed of the telomere repeat binding proteins POT1, TRF1 and TRF2, and their associated proteins TIN2, RAP1 and TPP1 (Liu et al. 2004, Palm and de Lange 2008, Xin et al. 2008, Giannone et al. 2010).

According to literature data, shelterin complex is essential in telomere capping so that telomeres that are severely or completely devoid of telomeric proteins are more prone to damages and/or become the target of frequent recombination (Baumann and Cech 2001, de Lange 2005, Shakirov et al. 2005, 2009, Xin et al. 2008, Palm and de Lange 2008, Giannone et al. 2010). At the same time, shelterin regulates telomere transcription, telomere silencing and telomere sister cohesion through the association of shelterin with other proteins or protein complexes (Giannone et al. 2010).

Due to the importance of the shelterin complex in the telomere functioning, it is very intriguing that this important set of proteins is absent in the studied arthropod genomes, including the aphid one. According to different essays performed both in animal and plants, shelterin complex has a exquisite specificity for the telomeric TTAGGG repeats due to the presence of multiple TTAGGG recognition folds in the complex (de Lange 2005). The TTAGGG motif prevails in all multicellular animals, except round worms and arthropods, and is probably ancestral for all Metazoa (Traut et al. 2007). In arthrodopds the derived TTAGG motif has been evolved (Traut et al. 2007, Mandrioli et al. 2012). As a whole, a plausible scenario is that the shift to the TTAGG telomeric sequence negatively affected the binding of shelterin proteins to the single-strand G-rich telomeric DNA bringing to the loss of the shelterin genes in arthropods.

Exceptions to the presence of all the shelterin proteins have been already reported in literature since, for instance, the subunit TIN2 and TPP1 have been so far only found in vertebrates (de Lange 2005). At the same time the yeast *Saccharomyces cerevisiae* Meyen, 1883 lacks the TRF-like protein and uses instead a highly diverged Rap1 orthologue that binds double-stranded telomeric DNA (de Lange 2005). Conversely, yeast telomeres contain Rif1, a conserved protein that has no known role at mammalian telomeres and instead functions in the intra-S-phase checkpoint (Silverman et al. 2004).

The absence of the whole shelterin complex is extremely interesting from a functional point of view since it is generally implicated in the generation of the t-loop and in the control of the synthesis of telomeric DNA by telomerase (de Lange 2005). According to the reported role for the shelterin complex, it could be interesting to better understand how t-loops can be generated in the absence of TRF1 and POT1. Interestingly, the availability of antibodies against G-quadruplex DNA (Schaffitzel et al. 2010) could allow to use them as a specific probe to identify and study the interaction of the telomere end-binding proteins with the G-quadruplex in different arthropods (including aphids) making possible to go in depth in the study of arthropod telomere functioning.
